# BRCA1 controls the cell division axis and governs ploidy and phenotype in human mammary cells

**DOI:** 10.18632/oncotarget.15688

**Published:** 2017-02-25

**Authors:** Zhengcheng He, Nagarajan Kannan, Oksana Nemirovsky, Helen Chen, Marisa Connell, Brian Taylor, Jihong Jiang, Linda M. Pilarski, Markus C. Fleisch, Dieter Niederacher, Miguel Angel Pujana, Connie J. Eaves, Christopher A. Maxwell

**Affiliations:** ^1^ Department of Pediatrics, University of British Columbia, Vancouver, British Columbia, Canada; ^2^ Terry Fox Laboratory, British Columbia Cancer Agency, Vancouver, British Columbia, Canada; ^3^ Department of Laboratory Medicine and Pathology, Division of Experimental Pathology and Laboratory Medicine, Mayo Clinic, Rochester, MN, USA; ^4^ Department of Oncology, University of Alberta and Cross Cancer Institute, Edmonton, Alberta, Canada; ^5^ Department of Obstetrics and Gynaecology, Landesfrauenklinik, HELIOS University Medical Center, Wuppertal, Germany; ^6^ Department of Gynaecology and Obstetrics, University Hospital Düsseldorf, Heinrich-Heine University Düsseldorf, Germany; ^7^ Breast Cancer and Systems Biology Unit, Program Against Cancer Therapeutic Resistance (ProCure), Catalan Institute of Oncology, IDIBELL, L’Hospitalet del Llobregat, Barcelona, Spain; ^8^ Department of Medical Genetics, University of British Columbia, Vancouver, British Columbia, Canada; ^9^ Michael Cuccione Childhood Cancer Research Program, BC Children’s Hospital, Vancouver, British Columbia, Canada

**Keywords:** BRCA1, spindle orientation, mitotic instability, human mammary epithelial cells

## Abstract

*BRCA1* deficiency may perturb the differentiation hierarchy present in the normal mammary gland and is associated with the genesis of breast cancers that are genomically unstable and typically display a basal-like transcriptome. Oriented cell division is a mechanism known to regulate cell fates and to restrict tumor formation. We now show that the cell division axis is altered following shRNA-mediated BRCA1 depletion in immortalized but non-tumorigenic, or freshly isolated normal human mammary cells with graded consequences in progeny cells that include aneuploidy, perturbation of cell polarity in spheroid cultures, and a selective loss of cells with luminal features. BRCA1 depletion stabilizes HMMR abundance and disrupts cortical asymmetry of NUMA-dynein complexes in dividing cells such that polarity cues provided by cell-matrix adhesions were not able to orient division. We also show that immortalized mammary cells carrying a mutant *BRCA1* allele (*BRCA1 185delAG/+*) reproduce many of these effects but in this model, oriented divisions were maintained through cues provided by CDH1^+^ cell-cell junctions. These findings reveal a previously unknown effect of BRCA1 suppression on mechanisms that regulate the cell division axis in proliferating, non-transformed human mammary epithelial cells and consequent downstream effects on the mitotic integrity and phenotype control of their progeny.

## INTRODUCTION

*BRCA1* deficiency is associated with the genesis of breast cancers that are genomically unstable and typically display a basal-like transcriptome [1], thus resembling the cells that constitute the outer “basal” layer of the normal adult human mammary gland. This compartment of the normal gland contains primitive cells able to regenerate normal-appearing, bilayered mammary structures *in vivo* and colonies *in vitro* that contain cells with variable numbers of luminal as well as basal features [2, 3]. While a number of studies have suggested that BRCA1 deficiency perturbs the differentiation hierarchy present in the normal mammary gland [4–6], little is known about the mechanisms that might explain a link between the altered differentiation, proliferation control and genomic instability characteristic of transformed BRCA1-deficient mammary epithelial cells.

In several tissues, including the mammary gland [7], oriented cell division is one mechanism which cells use to generate genomically identical but functionally distinct daughter cells. Tricellular junctions in epithelium serve as polarity cues [8] and adhesive cues from the microenvironment exert force along the retraction fibers that orient the mitotic spindle [9]. These external forces are integrated with dynein motor forces that are anchored to the cortex of dividing cells through LGN-NUMA complexes [10] and if the formation of these anchoring complexes is disrupted, the orientation of the cell division axis becomes deregulated [10] with subsequent progeny exhibiting aneuploid phenotypes, such as micronuclei [11]. The location, content, and activity of these dynein complexes are established by biochemical gradients of Ran-GTP at chromosomes and polo-like kinase 1 (PLK1) at spindle poles [10] or kinetochores [12], as well as a spindle pole located complex of hyaluronan-mediated motility receptor (HMMR) and dynein light chain 1 [13].

BRCA1 has been reported to impact multiple critical nodes during the process of cell division. It is normally found at the mitotic spindle poles [14] and its deficiency is associated with genomic instability [15]. In immortalized cells and model systems, BRCA1 deficiency has been linked to centrosome dysfunction [16] and dysregulated mitotic spindle assembly [17] as well as genomic instability [18]. Mechanistically, BRCA1 reduces PLK1 activity [19] and forms mutually exclusive complexes with NUMA or HMMR to regulate Ran-dependent microtubule assembly [17] and promote the degradation of HMMR [16]. HMMR is also an upstream regulator for aurora kinase A [20, 21], which is known to influence symmetric division in mammary epithelial cells [22]. These tumor-suppressive actions may be specific for BRCA1, as polymorphisms in *HMMR* modify breast cancer risk associated with mutations in *BRCA1* but not *BRCA2* [23]. Thus, inappropriate orientation of cell division is a previously unexplored mechanism by which a loss or significant decrease in normal BRCA1 levels could alter the survival, growth, polarization and subsequent phenotypic characteristics of mammary cells. The present study was designed to address this hypothesis.

## RESULTS

### Suppression of BRCA1 randomizes the cell division axis

**Figure 1:**
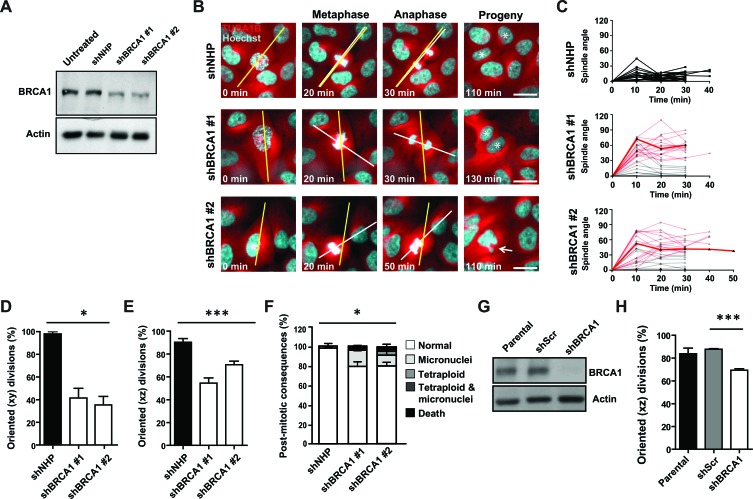
BRCA1 is required to orient the cell division axis **A**. Levels of BRCA1 protein measured in cell lysates of MCF-10A-TUBA1B-RFP cells obtained 2 days following transduction. Actin was used as a loading control. **B**. Mitotic spindle oscillations and cell division angles in MCF-10A-TUBA1B-RFP cells measured 3 days post-transduction. The long axis of the cell (yellow line) and mitotic spindle axis (white line) are indicated. Asterisks indicate progeny cells and the arrow indicates a progeny cell that underwent mitotic slippage and contains a micronucleus. Scale bars = 20 μm. **C**. The oscillation of the mitotic spindle tracked from metaphase through anaphase. Red lines indicate anaphase cells with cell division angles ≥30°. The highlighted red lines indicate the spindle oscillation seen in the shBRCA1-transduced cells shown in panel B. (*n* = 24, 24, or 22 cell divisions for NHP, shBRCA1#1, or shBRCA1#2). **D**. Percentage of oriented cell divisions (MCF-10A-TUBA1B-RFP cells) in the xy plane (*n* = 48, 48, or 46 cell divisions for NHP, shBRCA1#1, or shBRCA1#2). *P < 0.05; ANOVA. **E**. Percentage of oriented cell divisions (MCF-10A-TUBA1B-RFP cells) in the xz plane (*n* = 87, 34, or 83 cell divisions for NHP, shBRCA1#1, or shBRCA1#2). ***P < 0.0001; ANOVA. **F**. Frequency of post-mitotic consequences in MCF-10A-TUBA1B-RFP cells (*n* = 48, 48, or 46 cell divisions for NHP, shBRCA1#1, or shBRCA1#2). *P < 0.05; ANOVA. **G**. Levels of BRCA1 measured in lysates of MCF-12A sublines prepared following treatment with 2 μg/ml doxycycline for 96 hours to induce the expression of a scrambled shRNA or a shRNA targeting BRCA1. Actin was used as a loading control. **H**. Percentage of oriented cell divisions (MCF-12A cells) in the xz plane (*n* = 86, 58, or 70 mitotic cells analyzed for parental, scrambled shRNA, or shBRCA1). ***P < 0.0001; two-tailed unpaired t-test.

In a first set of experiments, we examined the effect of lenti-shRNA-mediated BRCA1 depletion in a subline of the non-tumorigenic, but immortalized, MCF-10A human mammary epithelial cell line that stably expresses a TUBA1B-RFP fusion protein from the endogenous *TUBA1B* locus (Figure 1A). The use of this model, in combination with Hoechst counterstaining of DNA, allows both the kinetics of cell division and the spindle architecture and orientation to be visualized simultaneously in real time (Figure 1B). Time-lapse microscopy starting 72 hours post-transduction showed the orientation of the mitotic spindle in most of the dividing control-transduced cells to be oriented parallel to the long axis of the cell (within 30°C, Figure 1B) and the plane of the substratum (Supplementary Figure 1A) as expected [13]. In control-treated cells, measurements of the oscillation of the metaphase spindle revealed the spindle to set up within 30 °C of the cell's long axis and remain relatively fixed at this angle until anaphase (Figure 1C); indeed, nearly all metaphase spindles were oriented, which was defined as within 30°C of the long axis measured in the xy plane (Figure 1D) or to the plane of the substratum measured in the z-plane (Figure 1E). In contrast, the angle of the spindle was not restricted to within 30 °C of the cell's long axis in *BRCA1*-shRNA-transduced cells (Figures 1C - 1E), and the progeny of these divisions exhibited a significant increase in the frequency of micronuclei, mitotic slippage and exit with a tetraploid genome, or mitotic death (Figure 1F), despite the absence of any significant alteration in their division kinetics (Supplementary Figure 1B). Our examination of fixed aliquots of these cells 72 hours post *BRCA1-*shRNA transduction did not reveal changes to centrosome content or evidence of multipolar mitotic spindles (Supplementary Figures 1C - S1E), indicating that misoriented cell divisions occurred in the absence of these alterations.

In a next series of experiments, we examined the response of a second immortalized and non-tumorigenic human mammary epithelial cell line, MCF-12A cells, using a doxycycline inducible construct to reduce BRCA1 levels, as confirmed by Western blot analyses (Figure 1G). Fixed aliquots of these cells also showed a significant reduction in the frequencies of oriented cell divisions in the BRCA1-suppressed cells relative to controls (Figure 1H) without evidence of altered mitotic spindle architecture. Thus, BRCA1 is needed to position the spindle and restrict the division axis in non-tumorigenic human mammary cells.

### Suppression of BRCA1 alters the growth, phenotype and polarity of progeny cells

**Figure 2:**
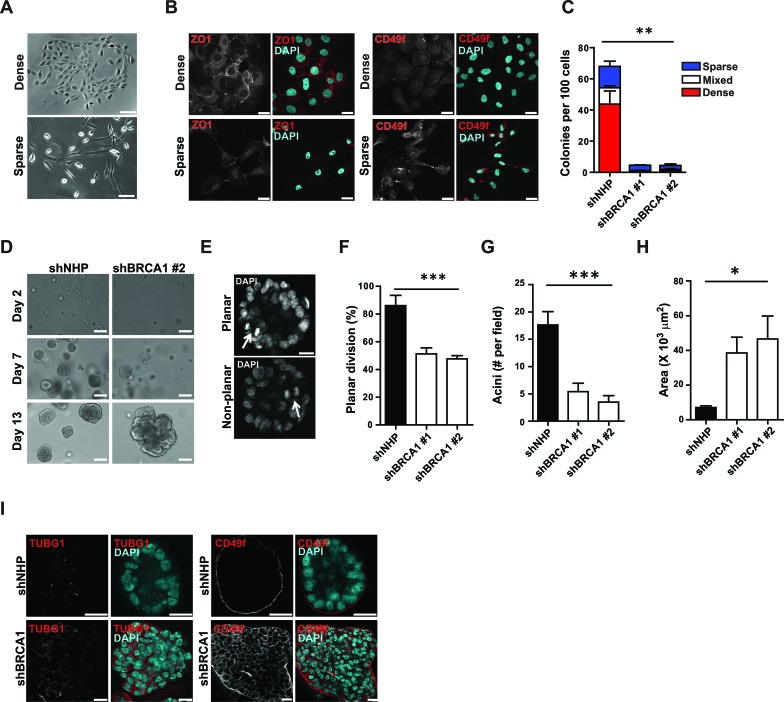
BRCA1 suppression reduces clonogenicity and alters phenotype and apicobasal polarity **A**. Bright field images of dense and sparse MCF-10A colonies. Scale bar = 100 μm. **B**. Detection of luminal (ZO1^+^) and basal (CD49f^+^) phenotypic markers in MCF-10A colonies. Scale bars = 20 μm. **C**. MCF-10A colonies displaying dense, sparse, or mixed phenotypes assessed 7 days post-transduction with virus encoding the indicated shRNA. **P < 0.001 for total and mixed colonies; *P < 0.05 for dense colonies; ns (*P* = 0.08) for sparse colonies; ANOVA. **D**. MCF-10A spheroids present after 2 days, or 1 or 2 weeks in culture after being transduced. Scale bars = 50 μm. **E**. Representative images of planar and non-planar cell division (arrows) during the second week in 3D cultures. Scale bar = 20 μm. **F**. Percentage of MCF-10A cells displaying planar cell division during growth in spheroid culture (*n* = 14, 64, or 60 cell divisions analyzed for NHP, shBRCA1#1, or shBRCA1#2). ***P < 0.0001; ANOVA. **G**. Mean number of acini per field of view (4X objective) measured during the second week of spheroid culture (*n* = 8, 7, or 4 fields of view for NHP, shBRCA1#1, or shBRCA1#2). ***P < 0.005; ANOVA. **H**. Acinar area measured during the second week of spheroid culture (*n* = 35, 38, or 53 acini for NHP, shBRCA1#1, or shBRCA1#2). *P < 0.05; ANOVA. **I**. Apical polarization of centrosomes (TUBG1^+^, left-hand side), and basal polarization of CD49f (right-hand side) measured in acini fixed during the second week of spheroid culture and counterstained with DAPI. Scale bars = 20 μm.

In standard 2D cultures, MCF-10A cells produce a mixture of cell types that are phenotypically similar to normal basal and luminal mammary epithelial cells [24] (Figures 2A, 2B). Therefore, we next asked whether an inability to restrict the cell division axis would affect the phenotypes of the progeny of such divisions. Accordingly, shRNA-transduced MCF-10A-TUBA1B-RFP cells were seeded at a low density and 5 days later, the number of colonies was counted, their morphology noted, and the cells within them examined by immunofluorescence. 68.8 ± 8.8% of the control-transduced cells were clonogenic and produced primarily dense colonies that consisted almost exclusively of cells expressing ZO-1^+^ junctions and low levels of CD49f (Figures 2A – 2C). Sparse colonies were less frequent and consisted mainly of cells expressing high levels of CD49f and few cell junctions (Figures 2A – 2C). In contrast, *BRCA1*-shRNA-transduction markedly reduced total clonogenic ability (>10-fold), with a selective loss of dense colonies of luminal-like cells (Figure 2C).

Oriented cell divisions are thought to establish and maintain the apicobasal polarized organization of epithelial cells [25]. It was therefore of interest to examine the effects of BRCA1 suppression on these properties in MCF-10A cells proliferating in 3D matrigel cultures. As expected, within 2 weeks, control-transduced MCF-10A cells formed polarized spheroids in matrigel (Figure 2D) with most of the mitotic cells at that time executing planar divisions (defined as ≤30° off the plane of the basal surface immediately below, Figures 2E and 2F). Again, *BRCA1*-shRNA-transduced cells formed fewer structures when plated at low density in matrigel (Figure 2G) but, the structures they did produce were larger (Figure 2H), irregular in shape, and lacked the apically polarized centrosomes and basally localized expression of CD49f (α6 integrin) characteristic of control spheroids (Figure 2I). Importantly, planar divisions were no longer prominent amongst the progeny that were continuing to divide 2 weeks later (Figure 2F). Together, these data indicate the cell division axis is not restricted following BRCA1 suppression and the immediate progeny of misoriented cell divisions demonstrate an associated reduction in clonogenic capacity and failure to acquire luminal features.

### Suppression of BRCA1 inhibits growth and disturbs the axis of division for primary normal human mammary epithelial cells

**Figure 3:**
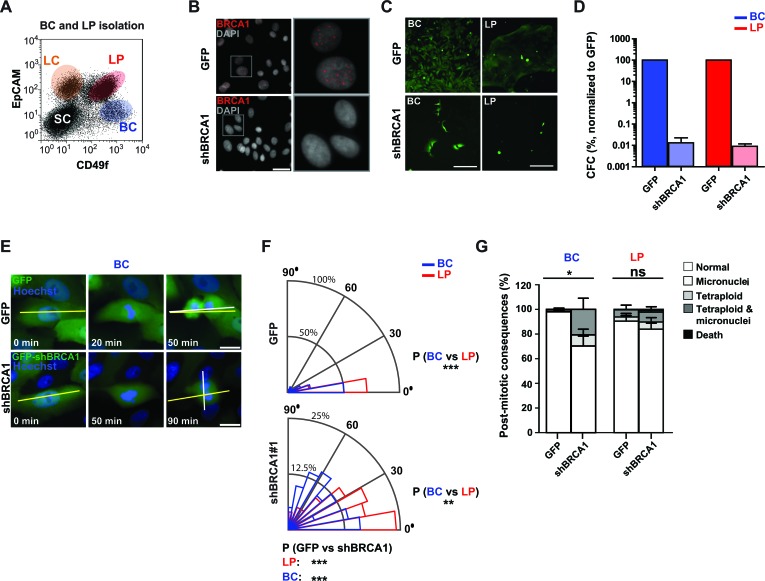
BRCA1 suppression disrupts the division axis in human mammary LPs and BCs isolated from non-disease human mammary tissue **A**. Flow cytometric profile showing gates used to isolate EpCAM^−/low^CD49f^+^ BCs and EpCAM^+^CD49f^+^ LPs. Also shown for reference are the gates that circumscribe the non-proliferative mammary luminal cells (LCs) and co-existing stromal cells (SCs). **B**. LPs were transduced with virus encoding GFP alone or a BRCA1 shRNA (shBRCA1) and GFP and the levels and localization of BRCA1 was assessed by immunofluorescence 3-5 days later. Scale bar = 40 μm. **C**. BCs or LPs were transduced and seeded at clonal density two days later. Images (cells from donor 8) of day 7 colonies indicate the proliferative capacity of the cells in 2D cultures. Scale bars = 200 μm. **D**. Clonogenic cell frequencies, normalized to that of control GFP-transduced cells, for BCs (blue, *n* = 2 donors) and LPs (red, *n* = 3 donors) transduced with shBRCA1#1. **E**. BCs transduced as indicated were imaged live for 10 to 24 hours at 10 minute intervals. The long axis of the cell (yellow line) and the cell division plane measured at anaphase (white line) are indicated. Scale bars = 20 μm. **F**. Circular graphs show the distribution of cell division angles measured at anaphase in 10°-wide sectors for BCs (blue) and LPs (red). Data for cells from the 8 donors shown individually is in Figure S2D (*n* = 109 cell divisions for BCs with GFP, *n* = 84 for BCs with shBRCA1#1 and GFP, *n* = 106 for LPs with GFP, and *n* = 82 for LPs with shBRCA1#1 and GFP). ***P < 0.0001; two-tailed unpaired *t*-test. **G**. Percentage of aberrant post-mitotic events in the immediate progeny of BCs (blue) and LPs (red) transduced as indicated. Data for cells from the 8 donors shown individually is in Figure S2E (*n* = 109 cell divisions for BCs with GFP, *n* = 84 for BCs with shBRCA1#1 and GFP, *n* = 106 for LPs with GFP, and *n* = 82 for LPs with shBRCA1#1 and GFP). *P < 0.05 or ns- *P* > 0.05; two-tailed paired *t*-test.

We next sought to test whether reducing BRCA1 levels would affect the mitotic behavior or integrity of freshly isolated normal human mammary progenitor cells. We have previously shown that normal breast tissue contains two phenotypically separable and functionally distinct progenitor cell types each constituting ~20%-30% of the cells in the phenotype used to distinguish them (Figure 3A). One is characterized by a basal cell (BC) phenotype and generates colonies of cells that are either exclusively myoepithelial mixed with luminal cells in 2D and 3D cultures. The other is referred to as luminal progenitors (LPs) and these generate colonies of luminal cells exclusively in either 2D or 3D cultures [3]. Transduction of purified fractions of these cells with our *BRCA1* shRNA or control vector led to suppression of BRCA1 expression by 1-2 days later (Figure 3B). The control-treated cells displayed expected clonogenic activity in both 2D assays (Figures 3C, 3D) and 3D assays (Supplementary Figures 2A and S2B), whereas the *BRCA1*-shRNA-transduction produced a pronounced loss of clonogenic activity in both the BC and LP fractions (Figure 3D) with no significant induction of multipolar spindle architecture (Supplementary Figure 2C). However, both phenotypes of progenitors showed a consistent increase in the frequency of divisions in which the normal axis was misoriented (≥30° off the long axis at anaphase, Figure 3E; spindle angle distribution across 8 donor samples shown in Figure 3F; individual donor sample data shown in Supplementary Figure 2D). As previously observed in the MCF-10A cells, time-lapse imaging of the transduced cells over a 24 hour period revealed that misoriented divisions often produced micronuclei and/or mitotic slippage but here with the effects being more pronounced in the *BRCA1-*shRNA transduced progeny of the BCs relative to their donor-matched LPs (post-mitotic consequences across 8 donor samples shown in Figure 3G; individual donor sample data shown in Supplementary Figure 2E). Marked BRCA1 suppression was so inhibitory to primary mammary cells that more subtle impacts on other programs could not be assessed. However, the more pronounced effects on the cell division axis observed in primary BCs may indicate a heightened reliance on BRCA1 function or the potential existence of compensatory mechanisms within donor-matched LPs.

### Suppression of BRCA1 disturbs cortical NUMA-dynein asymmetry and stabilizes HMMR

**Figure 4:**
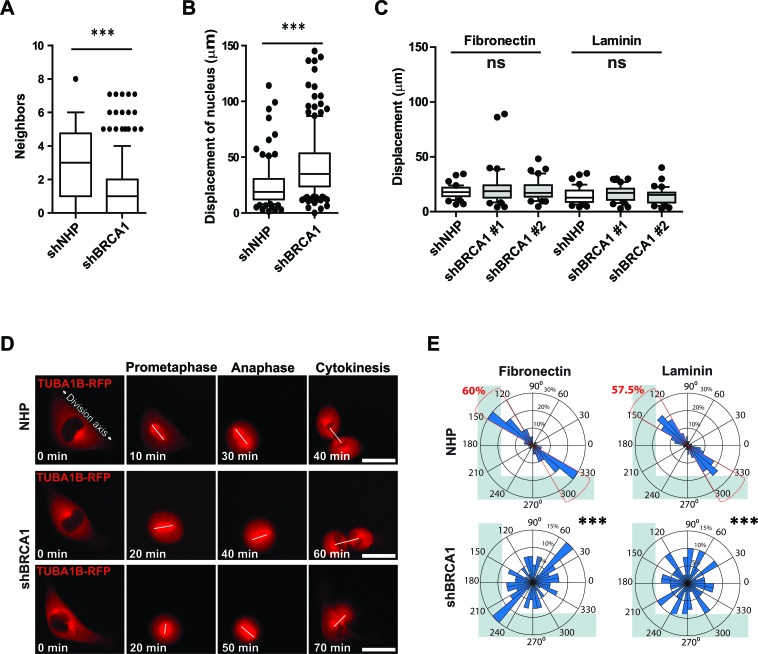
BRCA1 is required for intrinsic spindle positioning in isolated cells **A**. Neighboring cell numbers for mitotic MCF-10A-TUBA1B-RFP cells measured post transduction in day 7 colonies (*n* = 100 cell divisions for NHP and *n* = 200 cell divisions for shBRCA1#1&#2). Data presented as a box and whiskers (10 - 90 percentiles) plot. ***P < 0.0001; two-tailed unpaired t-test. **B**. Movement of mitotic MCF-10A-TUBA1B-RFP cells measured post-transduction in day 7 colonies (*n* = 100 mitotic cells for NHP and *n* = 180 mitotic cells for shBRCA1#1&#2). Data presented as a box and whiskers (10 - 90 percentiles) plot. ***P < 0.0001; two-tailed unpaired t-test. **C**. Movement, as indicated by the displacement of the nucleus, for mitotic MCF-10A-TUBA1B-RFP cells cultured on L-shaped micropatterns coated as indicated (n = 40 mitotic cells for each treatment). Data presented as a box and whiskers (10 - 90 percentiles) plot. Ns- P >0.05; ANOVA. **D**. MCF-10A-TUBA1B-RFP cells transduced and grown 72 hours later on L-shaped, fibronectin-coated micropatterns (see also Movie S1) with a white line drawn to connect the spindle poles. **E**. Circular graphs, superimposed on coated L-shaped micropatterns, show the distribution of cell division angles measured at anaphase (*n* = 40 cell divisions for each treatment). ***P < 0.0001; two-tailed unpaired t-test.

The cell division axis and the position of the mitotic spindle is responsive to multiple cues, including an intrinsic pathway that establishes cortical asymmetry of NUMA-dynein complexes [10] and positioning cues from cell-cell junctions [8] or cell-matrix adhesions [9]. Time-lapse imaging of MCF-10A colonies as they formed suggested cell-cell junctions and cell-matrix adhesion were altered for *BRCA1*-shRNA-transduced mitotic cells, which had fewer neighboring cells and underwent large movements during division (Figures 4A and 4B). To examine the influence of cell-matrix adhesions on the cell division axis and at the same time reduce the potential contribution of cell migration and remove cell-cell adhesions between neighboring cells, we next examined the division of individual control- and *BRCA1* shRNA- transduced cells on defined adhesive substrates. When grown on L-shaped laminin- or fibronectin-coated micropatterns (Movie S1), the displacement of dividing cells (Figure 4C), their kinetics of spreading (Supplementary Figures 3A), and the formation of F-actin- or vinculin^+^ adhesive structures (Supplementary Figures 3B) was similar between control-treated and BRCA1-depleted mitotic cells. In control-treated mitotic cells, adhesive cues (both fibronectin and laminin) fixed the orientation of the mitotic spindle and the division axis parallel to the hypotenuse of the L-shaped pattern (Figures 4D and 4E; Movie S1). In BRCA1-depleted cells, however, these spatially-restricted cell-matrix cues were not sufficient to orient or fix the mitotic spindle, which often rotated during division and resulted in an arbitrary division axis (Figures 4D and 4E; Movie S1). These data suggest a disruption of the establishment or maintenance of motor protein forces along the cortex of dividing BRCA1-depleted cells.

**Figure 5:**
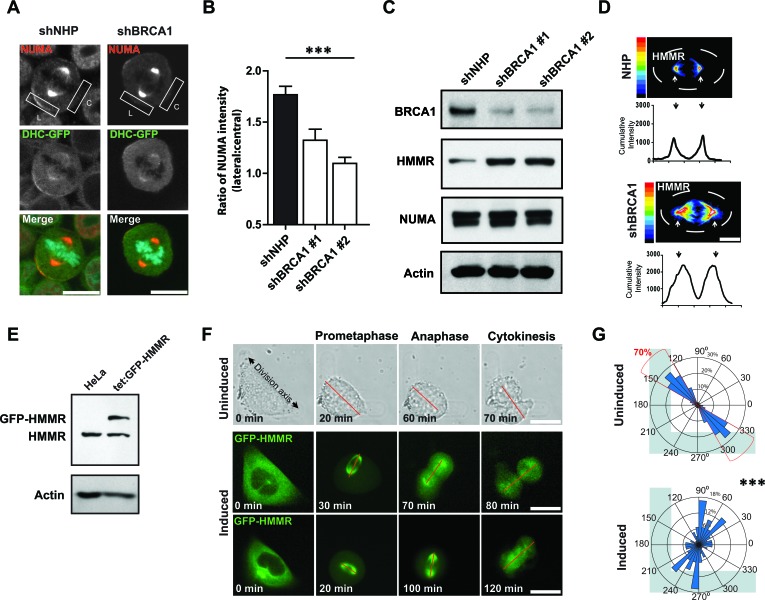
BRCA1 establishes cortical asymmetry of dynein motor complexes **A**. Immunofluorescence showing the localization of NUMA and dynein heavy chain (DHC)-GFP on the lateral (L) or central (C) cell cortex in DHC-GFP HeLa cells. Scale bars = 20 μm. **B.** Quantification of the data from panel H showing the ratio of NUMA intensity on the lateral and central cortex (*n* = 27, 19, or 30 mitotic cells for NHP, shBRCA1#1, or shBRCA1#2). ***P < 0.0001; ANOVA. **C**. Levels of BRCA1, NUMA, and HMMR proteins measured in lysates from MCF-10A-TUBA1B-RFP cells obtained 2 days following transduction. Actin was used as a loading control. **D**. Immunofluorescence heatmap graphs (upper) and intensity graphs (lower) showing HMMR localization and abundance in mitotic MCF-10A cells. Scale bar = 20 μm. **E**. Doxycycline treatment induces the expression of GFP-HMMR in HeLa cells. Actin levels confirmed equal loading. **F**. Cell division in uninduced cells or following doxycycline induction for GFP-HMMR HeLa cells dividing on fibronectin-coated, L-shaped micropatterns (see also Movie S2). A red line connects the spindle poles. Scale bars = 20 μm. **G**. Circular graphs superimposed on L-shaped micropatterns show the distribution of cell division angles measured at anaphase (*n* = 60 cell divisions for each treatment). ***P < 0.0001; two-tailed unpaired t-test.

The spindle positioning pathway establishes the asymmetric localization of dynein anchoring proteins, such as NUMA, and dynein complexes at the cortex of the dividing cell. So, we used a subline of HeLa cells that had been engineered to stably express a GFP-tagged dynein heavy chain to examine the effect of BRCA1 depletion on the subcellular distribution of cortical dynein and NUMA. Control-treated mitotic cells demonstrated the expected localization of NUMA and dynein heavy chain at lateral but not central regions of the cortex (Figures 5A and 5B). However, the asymmetric localization of NUMA was disturbed in the *BRCA1*-shRNA-transduced mitotic cells (Figure 5B), indicating a deficit in the intrinsic spindle positioning pathway. We next queried the effect of BRCA1 suppression on the abundance of HMMR and NUMA that, in turn, are known to regulate cortical dynein complexes [10, 13]. BRCA1 depletion did not affect NUMA levels in MCF-10A-TUBA1B-RFP cells but did stabilize HMMR levels, which were found to be augmented by Western analyses of mitotic lysates (Figure 5C) and along the mitotic spindles when examined by immunofluorescence (Figure 5D). To model the HMMR stabilization that results following BRCA1 depletion, we turned to a subline of HeLa cells in which the expression of mitotic spindle localized GFP-HMMR could be regulated by exposure to doxycycline (Figure 5E). Assessment of these cells when plated on L-shaped micropatterns showed their divisions recapitulated the same inability to establish a fixed, oriented spindle position and a defined division axis (Figures 5F and 5G; Movie S2). Thus, augmented expression of HMMR, as obtained following BRCA1 suppression or through inducible over-expression of GFP-HMMR, is sufficient to disrupt the intrinsic spindle positioning pathway.

### Cues from cell-cell junctions can overcome *BRCA1*-mutant effects on the division axis and phenotype control

**Figure 6:**
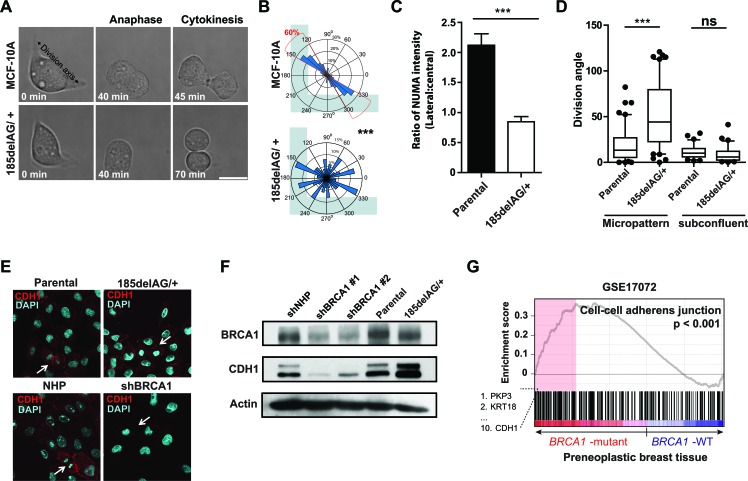
***BRCA1 185delAG/+*** exhibit deficient spindle positioning but orient cell division. **A**. MCF-10A cells (parental or *BRCA1 185delAG/+*) grown on fibronectin-coated, L-shaped micropatterns (see also Movie S3). **B**. Circular graphs superimposed on L-shaped micropatterns show the distribution of cell division angles measured at anaphase (*n* = 50 cell divisions for each treatment). ***P < 0.0001; two-tailed unpaired t-test. **C**. Quantification of NUMA intensity on the lateral and central cortex in mitotic *BRCA1 185delAG/+* or parental MCF-10A cells (*n* = 10 mitotic cells for parental and *n* = 20 mitotic cells for *BRCA1 185delAG/+*). ***P < 0.0001; two-tailed unpaired t-test. **D**. Cell division angles for anaphase cells grown on fibronectin-coated micropatterns or at subconfluent densities. Data presented as a box and whiskers (10 - 90 percentiles) plot (*n* = 60 cell divisions on micropatterns; *n* = 30 cell divisions in subconfluent cultures). ***P < 0.0001; two-tailed unpaired t-test. **E**. Localization and abundance of CDH1 in day 5 colonies of MCF-10A cells (parental or *BRCA1 185delAG/+*, control- or shBRCA1-transduced). Arrows indicate mitotic cells. **F**. BRCA1 and CDH1 levels in day 5 colonies of MCF-10A cells (parental or *BRCA1 185delAG/+*, control- or shBRCA1-transduced). Actin was used as a loading control. **G**. Gene set enrichment analysis (GSEA) graphical outputs for the association analysis of the expression differences between normal and pre-neoplastic *BRCA1* mutant breast tissue and genes annotated with the GO term “cell-cell adherens junctions” (GO:0005913). The enrichment score and p value are shown.

We then analyzed immortalized, non-malignant human mammary cells with a single knock-in allele encoding the pathogenic *BRCA1 185delAG* mutation [18] to more closely mimic the BRCA1+/− carrier state. Individual *BRCA1 185delAG/+* cells grown on fibronectin-coated, L-shaped micropatterns also showed a defective ability to orient their axis of cell division (Figures 6A and 6B; Movie S3; *hTERT*-IMECs, Supplementary Figure 4A) without noticeable alterations to cell-matrix adhesions (Supplementary Figure 4B). Consistently, the normal asymmetrically localization of cortical NUMA was disturbed in mitotic *BRCA1 185delAG/+* MCF-10A cells grown at a low density in regular 2D plates (Figure 6C). However, cell division angles (Figure 6D), clonogenic potential and progeny phenotypes were not altered (Supplementary Figure 4C), suggesting the deficient spindle positioning pathway observed for individual *BRCA1 185delAG/+* cells is overcome in these cultures. Cellular junctions in epithelium serve as polarity cues [8] and in cultures of *BRCA1 185delAG/+* MCF-10A cells, we noted CDH1^+^ cell-cell junctions and augmented CDH1 abundance relative to parental cells (Figures 6E and 6F). Moreover, genes classified as “cell-cell adherens junction” by gene ontology (GO), including *CDH1*, also showed elevated expression (Figure 6G) in pre-neoplastic mammary tissue from *BRCA1* mutant carriers [4]. Therefore, mutation of one *BRCA1* allele is sufficient to disturb the intrinsic spindle positioning pathway for individual cells but inadequate to randomize cell division angles or modify phenotype in subconfluent cells or those grown as colonies.

**Figure 7:**
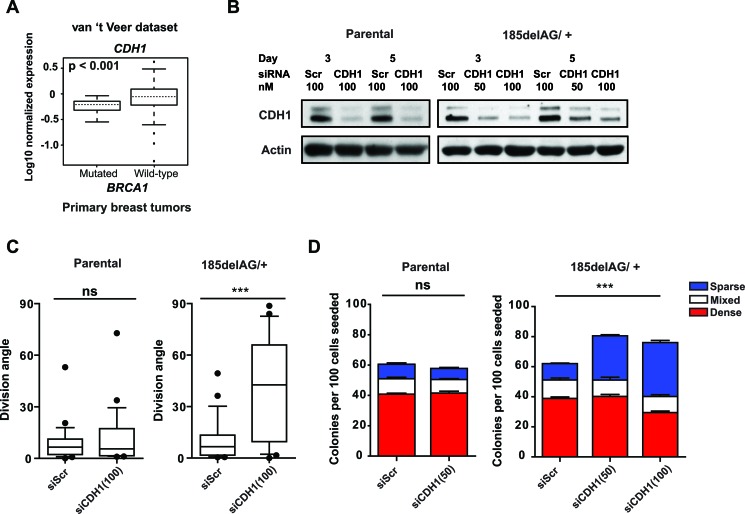
Cell junctional cues control cell division in ***BRCA1*** mutant cells. **A**. Box plot of *CDH1* expression levels in tumours with *BRCA1* mutations (*n* = 18) relative to those without (*n* = 80 downloaded from [27]). **B**. Levels of CDH1 in cell lysates of parental or *BRCA1 185delAG/+* MCF-10A cells obtained 3 and 5 days following transfection with either scrambled siRNA or siRNA targeting CDH1 (50 nM or 100 nM). Actin was used as a loading control. **C**. Cell division angles measured during anaphase in day 4 colonies derived from parental or *BRCA1 185delAG/+* MCF-10A cells transfected either with scrambled siRNA or siRNA targeting CDH1 (100 nM) (*n* = 25 cell divisions for each treatment). Data presented as a box and whiskers (10 - 90 percentiles) plot. ***P < 0.0001; two-tailed unpaired *t*-test. **D**. Percentage of parental or *BRCA1 185delAG/+* MCF-10A colonies at day 5 and displaying dense-, sparse-, or mixed- phenotypes following transfection with either scrambled siRNA or a siRNA targeting CDH1 (50 nM or 100 nM). ns for all comparisons in parental MCF-10A cells; ***P < 0.001 for total, dense, and sparse colonies, ns for mixed colonies in *BRCA1 185delAG*/+ MCF-10A cells; ANOVA.

*BRCA1* shRNA-transduced cells divide with arbitrary angles and we noted that *CDH1* was significantly downregulated in lysates prepared from these cells (Figure 6F) (also described for mammary cells that have been immortalized using *hTERT* [26]). Moreover, the normalized expression of *CDH1* is significantly reduced in tumours that arise in carriers of *BRCA1* mutations relative to sporadic breast tumors (Figure 7A) [27]. We speculated, therefore, that the attenuation or loss of positional cues provided through cell junctions may randomize division angles and influence colony phenotype in *BRCA1 185delAG/+* MCF-10A cells. To test this, we next examined the effect of suppressing CDH1 expression in the mutant and parental cells using a pool of siRNAs targeting *CDH1* (Figure 7B). The intact spindle positioning pathway correctly orients division for isolated MCF-10A cells (Figure 6B) and the suppression of CDH1 expression did not alter either cell division angles (Figure 7C) or colony phenotype (Figure 7D) in subconfluent cultures of these cells. The suppression of CDH1 expression in *BRCA1 185delAG/+* cells, however, significantly altered the cell division axis (Figure 7C) and augmented the production of progeny with basal-like features (Figure 7D). Thus, positional cues provided through cell-cell junctions are likely necessary to compensate for the defective intrinsic spindle positioning observed in *BRCA1 185delAG/+* MCF-10A cells; the suppression of CDH1+ positional cues combined with a deficient intrinsic spindle positioning results in arbitrary cell division angles with consequent alterations in the colony phenotypes produced from progeny of these divisions.

## DISCUSSION

The position of the mitotic spindle and the orientation of the division axis in many epithelial cells, including those of the mammary gland [7] relies on both intrinsic and extrinsic cues to maintain tissue architecture and planar cell divisions [28]. We have discovered a new and critical role of BRCA1 in the preservation of the cell division axis for proliferative, primary human mammary progenitor cells and immortalized, non-tumorigenic mammary cells. Our data reveal a cell-autonomous deficit in spindle positioning that disturbs the asymmetric localization of cortical NUMA-dynein motor complexes in BRCA1 suppressed and *BRCA1* mutant mammary cells, which results in an arbitrary cell division axis and resolves with progeny cells displaying aneuploid phenotypes, as also described following suppression of pVHL [29] or the disruption of NUMA-LGN complexes [11]. We show BRCA1 suppression augments HMMR abundance and the over-expression of GFP-HMMR is sufficient to disrupt the normal spindle positioning pathway. HMMR interacts with dynein adaptor proteins and through these complexes controls spindle position [13], which likely complements the actions of PLK1 and Ran-GTP [10]. Moreover, HMMR promotes PLK1 activity at kinetochores [20], which has been shown to strip LGN from the cortex when brought proximal on misaligned chromosomes [12]. Thus, it is reasonable to predict that PLK1 activity is augmented in BRCA1-depleted mitotic cells both through the stabilization of HMMR [20] and the removal of direct suppression of the kinase by BRCA1 [19]. Thus, loss of cortical NUMA-dynein complexes and a cell-autonomous deficit in spindle positioning observed following BRCA1 suppression is mechanistically explained by the stabilization of HMMR and augmented PLK1 activity predicted to occur along the spindle.

Given the multifunctional nature of BRCA1 and its potential to impact many critical nodes in the spindle positioning pathway, it is possible that the randomization of the cell division axis may result through the suppression of other BRCA1 functions, such as DNA damage repair [30], the NEAT1/ miRNA axis [31], or the RANK/ RANKL signaling pathway [32]. For example, caffeine-induced DNA damage and checkpoint abrogation misaligns chromosomes and induces asymmetric cell division in lymphocytes [33] while chromosome misalignment is also able to induce spindle-positioning defects [12]. Spindle-pole derived signals are critical to spindle positioning [10]; therefore, the induction of multipolar spindles through centrosome amplification would be predicted to disturb these positioning gradients. Centrosome amplification has been observed following BRCA1 suppression [16, 34] and in tetraploid cells following mitotic slippage or cytokinesis failure [35]. In our study, centrosome amplification did not precede the induction of an arbitrary division axis. But, we did note an increased frequency of mitotic slippage following misoriented divisions of BRCA1-silenced cells, which produced daughter cells exhibiting micronuclei, polypoidy, and growth inhibition as has been reported for chemical induction of slippage [36]. Thus, we do not exclude the potential for centrosome amplification to occur following mitotic slippage and, thus, the potential for other pathways that may be disturbed through BRCA1 suppression to contribute to spindle mispositioning.

Cell-matrix adhesive cues were not sufficient to overcome the intrinsic positioning deficit observed in *BRCA1 del185AG/+* cells. However, these *BRCA1* mutant cells orient their division axis when clonally expanded and generate colonies that express elevated levels of *CDH1*, which we also observed in pre-neoplastic mutant *BRCA1* relative to control mammary tissues [4]. Moreover, we find that CDH1 is required for oriented division in *BRCA1 del185AG/+* cells and its suppression differentially affect the basal and luminal programs. Thus, it might be speculated that mutant *BRCA1* function may have different effects in different mammary progenitor states *in vivo* [4, 6] based at least in part on the expression of CDH1.

Marked BRCA1 suppression was so inhibitory to primary mammary cells that more subtle impacts on other programs could not be assessed. However, elevated *CDH1* expression in luminal cells might be anticipated to counteract an inherent positioning deficit and provide these cells an advantage in mutant *BRCA1* mammary tissue. Such a postulate is supported by the report that the LP fraction is proportionally increased in nonmalignant mammary tissue obtained from mutant *BRCA1* carriers [4] and our observations of more pronounced effects in the *BRCA1-*shRNA transduced BCs relative to their donor-matched LPs. Primary BCs may lack cell-cell junctional cues needed to establish the division axis and, thus, be more highly reliant on the intrinsic positioning pathway and on the function of BRCA1. An elevated dependence of primary BCs on BRCA1 may also be linked to their inherently different cell shape [37] or dimensionality [38]. The present findings would thus add to previous evidence of a greater ability of LPs to survive mutagenic events than BCs [3, 39] and hence contribute to an increased susceptibility to transformation.

In summary, oriented cell division is a mechanism known to regulate cell fates and to restrict tumor formation. BRCA1 regulates mammary cell behavior by numerous mechanisms, including the response to DNA damage, transcription, and mitotic spindle assembly, and we now show that BRCA1 controls mitotic spindle positioning and dictates the cell division axis in partnership with cell junctional cues. Moreover, immediate consequences of BRCA1 suppression include a randomized cell division axis that elevates the frequency of progeny cells that display aneuploid phenotypes and fail to acquire luminal features. These findings help to explain how *BRCA1* mutation may perturb the differentiation hierarchy present in the normal mammary gland and why it is associated with the genesis of breast cancers that are genomically unstable and typically display a basal-like transcriptome.

## MATERIALS AND METHODS

Ethics approval and consent to participate: Tissue was obtained from consenting donors and used according to protocols approved by the University of British Columbia Research Ethics Board.

### Reagents and cell lines

The following antibodies were used: Actin (Sigma), BRCA1 (Cell Signaling, Western blot; clone SD118, Calbiochem, immunofluorescence), CD49f (Millipore), GFP (Abcam ab1218), beta-tubulin (TUBB)-Alexa Fluor 647 (Cell Signaling), gamma-tubulin (TUBG1) (Sigma), ZO1 (Invitrogen), CDH1 (Cell Signaling), Alexa fluor 488 phalloidin (Life Technologies) and secondary antibodies conjugated to horse radish peroxidase (Sigma) or conjugated to AlexaFluor647, 594, or 488 (Invitrogen).

MCF-10A cells were obtained directly from Dr. J. Brugge (Harvard University, Boston, MA). MCF-10A-TUBA1B-RFP cells with RFP-tagged TUBA1B were purchased from Sigma-Aldrich (CLL1039). Parental or *BRCA1 185delAG/+ hTERT*-IMECs and parental or *BRCA1 185delAG/+* MCF-10A were purchased from Horizon Discovery.

MCF-12A cells were purchased from ATCC. Cells were transduced with lentiviral particles containing Tet Repressor protein- (pLV-tTR-KRAB-IP) and shRNA-coding (pLV-THM) cassettes to create tet-on inducible expression of short hairpin RNAs (shRNA). One subline expressing a shRNA containing a scrambled sequence (shScr) with no target sequence in the human genome, served as a negative control for another subline that expressed a shRNA targeting BRCA1. Cells were treated with 2 μg/ml doxycycline for four days.

HeLa cells with mouse DHC-GFP were obtained from the Mitocheck consortium. HeLa cells with tet-on inducible expression of enhanced GFP fused in frame with full-length HMMR (GFP-HMMR) were produced from HeLa Tet-on cells (Clontech). Tet-on GFP-HMMR cells were cultured in the presence of 400 μg/ml G418 and 200 μg/ml hygromycin B (Sigma-Aldrich) for selection. GFP-RHAMM expression was induced with 2 μg/ml doxycycline.

### Isolation and culture of primitive human mammary cells

Highly purified subpopulations of human mammary epithelial cells were isolated from normal reduction mammoplasty samples as previously described [2, 3]. Briefly, this involved dissociating viably cryopreserved organoid preparations into single cell suspensions, and then isolating viable (DAPI^−^) EpCAM^−/low^CD49f^+^ basal cells (BCs) and EpCAM^+^CD49f^+^ LPs using cell sorting gates that excluded hematopoietic (CD45^+^), endothelial (CD31^+^), dead (DAPI^+^) cells and debris. Tissue was obtained from consenting donors and used according to protocols approved by the University of British Columbia Research Ethics Board.

### Cloning, lentivirus production, transduction, and transfection

Sequences encoding short hairpins against BRCA1 (shBRCA1 #1, shBRCA1 #2) or non-hairpin (shNHP) sequences were produced and used to transduce cells as described [23]. On-target plus siRNA (SMARTpool) targeting human CDH1 was purchased from Dharmacon, and scrambled siRNA was used as a negative control. Transfections of siRNA used Lipofectamine 3000™ (Invitrogen) following the manufacturer's protocols.

### Cell culture assays and western blot analysis

Following transduction, immortalized mammary epithelial cell lines were seeded into 6- or 96- well plates at 2-20 cells/cm^2^ and cultured for 7 days prior to scoring colonies containing ≥50 cells. MCF-10A cells were also cultured in matrigel [23]. Transduced primary human mammary cells were either cultured in 6 well plates under progenitor assay conditions or in matrigel (Gibco)-coated 96-well Nunc plates for live-imaging or embedded in matrigel and cultured for 10-12 days to obtain 3D structures [3]. Western blot analyses were performed as described [23].

### Microscopy

For studies of living cells, lentivirus transduced cells were seeded in 96 well plates and incubated overnight then stained with Hoechst (1 μg/μl). Cells were imaged every 5, 10, or 15 minutes for up to 24 hours at 37°C in a 5% CO_2_ environmental chamber using an ImageXpress Micro High Content Screening System (Molecular Devices Inc.). For experiments involving micropatterns (CYTOO), plates were coated either with fibronectin or laminin and 3,000 cells were seeded at a density of 15,000 cells/ml.

For confocal microscopy, fixed cells were imaged using a 60x oil objective with a 1.2 numerical aperture on an Olympus Fluoview FV10i (Olympus) confocal microscope. Image z-stacks consisted of 3 to 25 optical sections with a spacing of 1.0 μm through the cell or acinar volume. Images were processed and analysed using the Olympus Fluoview software.

For immunofluorescence analyses, cells were fixed, blocked, and antibodies were incubated and washed as described [23]. Coverslips were mounted with ProLong Gold Antifade Reagent containing DAPI (Invitrogen). Cells grown in matrigel were fixed in cold methanol for 30-60 minutes.

### Cell division axis and mitotic spindle orientation analyses

Adherent, living cells (x,y plane) were imaged, mitotic cells identified, and the long axis of G_2_ phase cells was determined 10 minutes prior to mitosis onset (defined by chromosome condensation). This plane then served as the vector relative to which the cell division plane was measured in anaphase cells. To assess cells dividing in 2D cultures (z plane), the cells were fixed in methanol and stained for TUBG1 and/or TUBB with DAPI. Metaphase, anaphase and telophase cells, but not abnormal mitotic spindles, were analyzed. Z-stacks were imaged at 1 μm steps and 3D reconstructions were created using Olympus software. To measure the angle of the mitotic spindle relative to the growth surface 3D reconstructions were rotated 90° [13]. In matrigel cultures, spheroids consisting of ≥50 cells, as approximated by nuclear DAPI staining, were stained and 3D reconstructions were generated. Mid-sections obtained from z-stacks were used to assess the cell division axis relative to the basal surface in metaphase, anaphase and telophase cells, the apical positioning of the centrosomes, and the basal deposition of CD49f. Abnormal mitotic spindles were excluded from the analysis. Based upon the angles of control-treated acini, a cut off of ≤30° was set for planar spindle orientation.

### Gene expression analyses

Pre-processed and normalized RNA-seq data was downloaded from the corresponding publication [27]. Normalized data from pre-neoplastic breast tissue was downloaded from the Gene Expression Omnibus reference GSE17072 [4]. The GSEA tool was used was run using default values for all parameters.

### Statistics

Data are expressed as mean ± standard error of the mean. Statistical analysis was performed using the unpaired two-tailed Student's t-test or an unpaired one-way ANOVA followed by Tukey's Multiple Comparison test with the following exceptions: paired two-tailed Student's t-test was used for comparison of primary cell data, including multipolar mitosis, and mitotic outcomes. The results were considered significant at *P* < 0.05.

## SUPPLEMENTARY MATERIALS FIGURES AND MOVIES








