# Supplementary material for: BRCA1 controls the cell division axis and governs ploidy and phenotype in human mammary cells
Source: Oncotarget. 2017 Feb 25;8(20):32461–75. doi: 10.18632/oncotarget.15688 (PMC5464802; doi:10.18632/oncotarget.15688)
Supplement: Supplementary file 1 [file oncotarget-08-32461-s001.pdf]

BRCA1 controls the cell division axis and governs ploidy and phenotype in human mammary cells

Supplementary Material

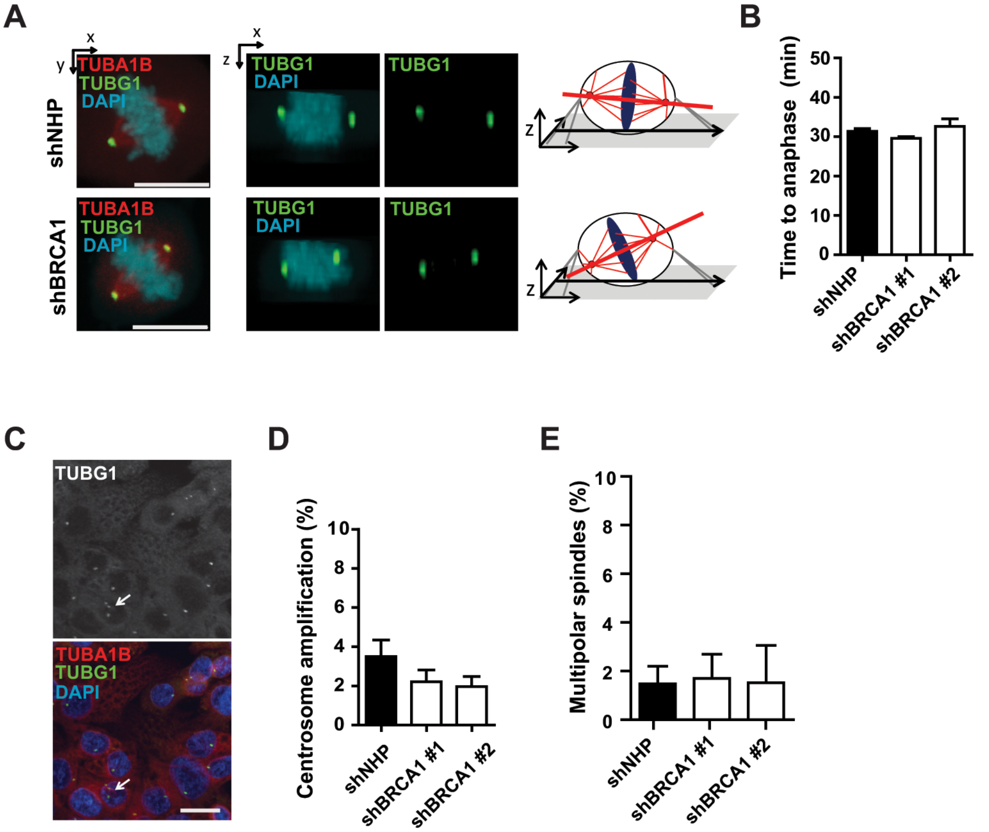

Figure S1 - Reduction of BRCA1 expression preserves kinetics and centrosome numbers during the initial cell divisions. (A) MCF10A-TUBA1B cells were transduced, fixed 3 days later, and confocal images were collected to create 3D reconstructions from metaphase cells. Slices were rotated 90° and the angle of a vector connecting the spindle poles (delineated by TUBG1) relative to the growth surface was measured. Scale bars = 10 µm. Model (right hand side) depicts the measurement of mitotic spindle angle with respect to the coverslip in adherent metaphase cells.

(B) The time needed for MCF-10A-TUBA1B-RFP cells to transit from prometaphase (indicated by chromosome condensation) through to the initiation of anaphase is shown (n=24, 24, or 22 cell divisions for NHP, shBRCA1#1, or shBRCA1#2).

(C) TUBG1^{+} centrosomes imaged in MCF-10A-TUBA1B-RFP cells transduced with shBRCA1#2. Arrow shows a cell with 3 centrosomes. Scale bar = 20 µm.

(D) The percentage of cells with greater than 2 centrosomes, termed centrosome amplification, is graphed for the mean ± SEM of three experiments (n=463, 754, or 885 cells analyzed for NHP, shBRCA1#1, or shBRCA1#2).

(E) The percentage of cells with greater than 2 spindle poles, termed multipolar spindles, is graphed for the mean ± SEM of three experiments (n=157, 177, or 113 mitotic cells analyzed for NHP, shBRCA1#1, or shBRCA1#2).

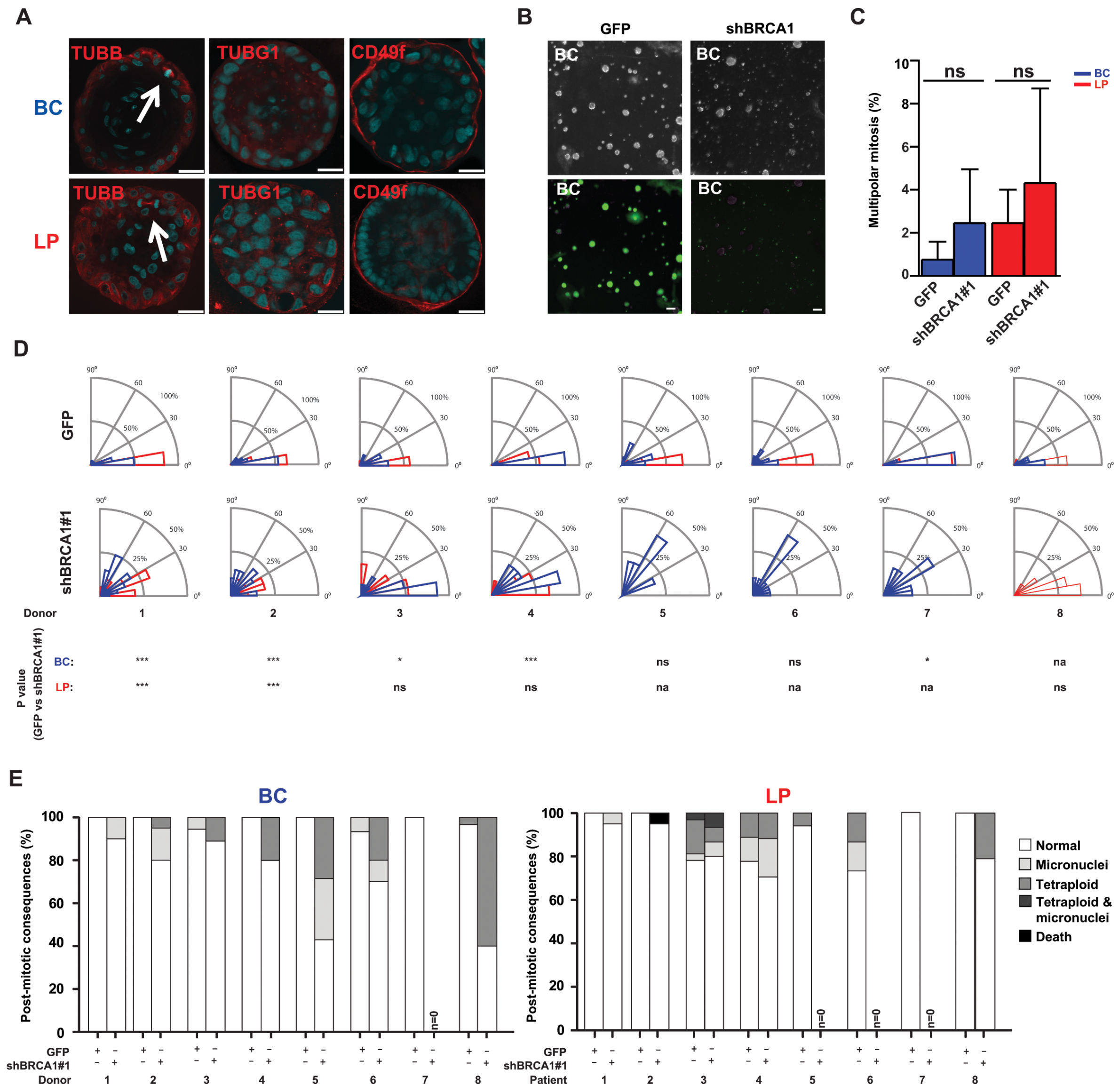

Figure S2 - Spheroid growth, division angles, and mitotic outcomes for normal human mammary BCs and LPs expressing GFP-control or BRCA1-shRNA.

(A) Representative images of lumen formation and overall spheroid morphology in matrigel cultures of BCs or LPs that were fixed and stained for mitotic spindles (TUBB, arrows indicate planar divisions), centrosomes (TUBG1), and/or CD49f (normally on the basal surface), and counterstained with DAPI. Scale bars = 20 µm.

(B) BCs or LPs were transduced with virus encoding GFP alone or shBRCA1 and GFP and seeded at clonal density in matrigel two days later. 10 days later fluorescent images (donor 8) representative of three donors indicate the proliferative capacity. The few acini that formed in the shBRCA1 treated gels did not express GFP, which is co-expressed with shBRCA1. Scale bars = 200 µm.

(C) Percentage of primary human mammary mitotic cells in 2D cultures with more than two spindle poles within 24 hours (n=109, 84, 106 or 82 mitotic cells for BCs with GFP, BCs with shBRCA1#1 and GFP, LPs with GFP, LPs with shBRCA1#1 and GFP). Ns- $P > 0.05$ by two-tailed paired t-test.

(D) Cell division angles in anaphase cells generated in 2D cultures from LPs (red) and BCs (blue) transduced as indicated. Data is presented separately for individual donor samples for which at least 5 mitotic cells were captured for the indicated treatment (numbers of mitotic cells analyzed for each patient listed as donor #, BCs with GFP, BCs with shBRCA1#1 and GFP, LPs with GFP, LPs with shBRCA1#1 and GFP: #1, 20, 20, 20, 20; #2, 20, 20, 20, 20; #3, 15, 16, 19, 11; #4, 13, 5, 9, 12; #5, 12, 5, 13, na; #6, 10, 10, 10, na; #7, 6, 6, 5, na; #8, 13, na, 10, 20). ***$P < 0.0001$, *$P < 0.05$, or ns- $P > 0.05$; two-tailed unpaired t-test. (na) indicates <5 mitotic cells were captured for one of the comparison populations.

(E) Post-mitotic outcomes for LPs and BCs transduced and cultured under 2D conditions as indicated (numbers of mitotic cells analysed for each patient is same as panel C). Data is presented separately for individual donor samples. N=0 indicates <5 mitotic cells were captured for that treatment condition.

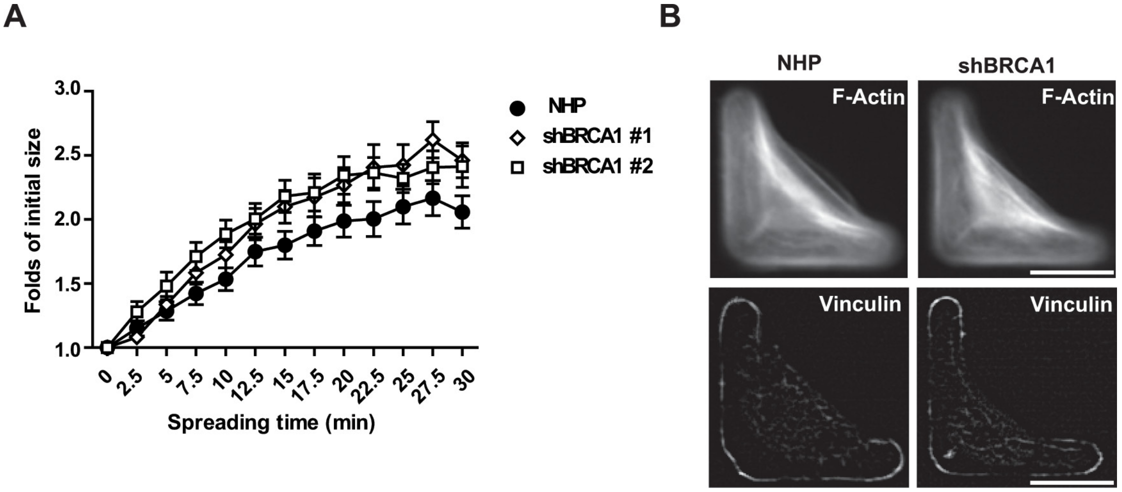

Figure S3 - Mitotic cell displacement and adhesions are normalized when grown on L-shaped micro-patterns.
(A) Cell spreading for MCF-10A-TUBA1B-RFP cells plated on L-shaped micropatterns coated with fibronectin and assessed 3 days after transduction (n=20 cells for each treatment). Data presented as mean ± SEM.
(B) F-actin and vinculin in MCF-10A-TUBA1B-RFP cells plated on L-shaped micropatterns coated with fibronectin and assessed 3 days after transduction. Scale bars are 20 μm.

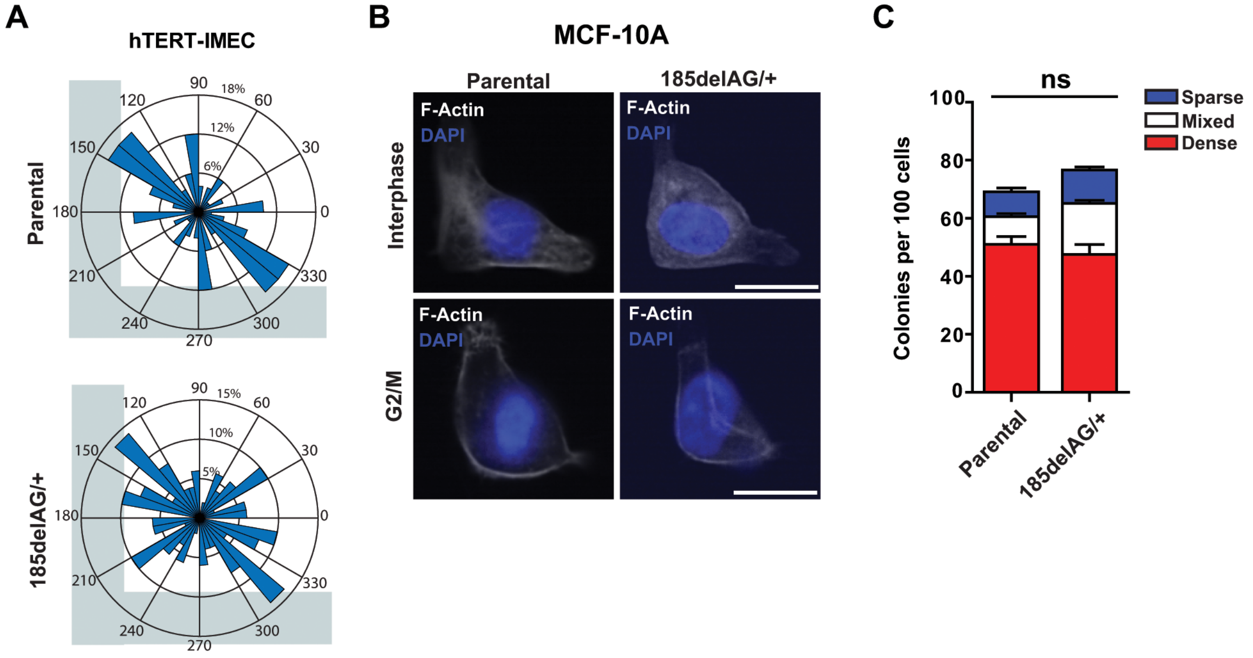

Figure S4 - BRCA1 185delAG/+ immortalized mammary epithelial cells misorient division but derive luminal-like colonies.

(A) Circular graphs superimposed on L-shaped micropatterns show the distribution of cell division angles measured at anaphase in 10°-wide sectors for parental or *BRCA1 185delAG/+ hTERT*-IMECs (n=50 cell divisions for each treatment).

(B) F-actin in parental or *BRCA1 185delAG/+* MCF-10A cells during interphase and G2/M phase when grown on L-shaped micropatterns. Scale bars = 20 μm.

(C) Number (per 100 cells plated) and phenotype of parental or *BRCA1 185delAG/+* MCF-10A cells assessed in day 5 colonies. ***P <0.001 for mixed colonies, ns for total, dense, and sparse colonies in *BRCA1 185delAG/+* MCF-10A cells; ANOVA.

Movie S1 - The orientation of the mitotic spindle is fixed and the cell division axis is strongly biased to occur along the hypotenuse of L-shaped, fibronectin-coated micropatterns for a MCF-10A-TUBA1B-RFP cell transduced 72 hours previously with lentivirus encoding non-hairpin shRNA (left-hand side). The orientation of the mitotic spindle is not fixed and the cell division axis is random relative to the L-shaped, fibronectin-coated micro-patterns for MCF-10A-TUBA1B-RFP cells transduced 72 hours previously with lentivirus encoding shRNA targeting BRCA1 (middle and right-hand side). Cells were followed through mitosis by time-lapse imaging for 1– 1.5 hours with images captured at 10 minute intervals. Cell division is shown through time at 2 frames per second. Red line indicates the anticipated cell division axis.

Movie S2 - The cell division axis is strongly biased along the hypotenuse of L-shaped, fibronectin-coated micropatterns for uninduced HeLa cells that express GFP-HMMR in a doxycycline responsive manner (left-hand side). The orientation of the mitotic spindle is not fixed and the cell division axis is random relative to the L-shaped, fibronectin-coated micropatterns following 8 hours incubation with 2μg/ml doxycycline (middle and right-hand side). Cell division was followed by time-lapse imaging for 1.5-2.5 hours with images captured at 10 minute intervals. Cell division is shown through time at 2 frames per second. Red line indicates the anticipated cell division axis.

Movie S3 - The cell division axis is strongly biased along the hypotenuse of L-shaped, fibronectin-coated micropatterns for a parental MCF-10A cell (left-hand side). However, the cell division axis is random relative to the L-shaped, fibronectin-coated micropatterns for a *BRCA1 185delAG/+* MCF-10A cell (right-hand side). Cells were followed through division by time-lapse imaging for 1.25 hours with images captured at 5 minute intervals. Cell division is shown through time at 2 frames per second. Red line indicates the anticipated cell division axis.
